# Design Optimization and Non-Linear Buckling Analysis of Spherical Composite Submersible Pressure Hull

**DOI:** 10.3390/ma13112439

**Published:** 2020-05-26

**Authors:** Muhammad Imran, Dongyan Shi, Lili Tong, Hafiz Muhammad Waqas, Riaz Muhammad, Muqeem Uddin, Asghar Khan

**Affiliations:** 1 College of Mechanical and Electrical Engineering, Harbin Engineering University, Harbin 150001, China; mimran@hrbeu.edu.cn (M.I.); hafizwaqas@hrbeu.edu.cn (H.M.W.); asghar81@hrbeu.edu.cn (A.K.); 2 College of Aerospace and Civil Engineering, Harbin Engineering University, Harbin 150001, China; tonglili@hrbeu.edu.cn; 3 Department of Mechanical Engineering, College of Engineering, University of Bahrain, Zallaq 1054, Kingdom of Bahrain; rmuhammad@uob.edu.bh; 4 Pakhtunkhwa Energy Development Organization (PEDO), Peshawar 25000, Pakistan; eng.muqim@gmail.com

**Keywords:** optimization, genetic algorithm, composite spherical pressure hull, static structural analysis, buckling analysis, collapse depth, imperfection sensitivity

## Abstract

This paper describes an optimization study of a spherical composite submersible pressure hull employing a genetic algorithm (GA) in ANSYS. A total of five lay-up arrangements were optimized for three unidirectional composites carbon/epoxy, glass/epoxy, and boron/epoxy. The minimization of the buoyancy factor (B.F) was selected as the design optimization objective. The Tsai-Wu and Tsai-Hill failure criteria and buckling strength factor (λ) were used as the material failure and instability constraints. To determine the effect of geometric non-linearity and imperfections on the optimized design, a non-linear buckling analysis was also carried out for one selected optimized design in ABAQUS. The non-linear buckling analysis was carried out using the modified RIKS procedure, in which the imperfection size changed from 1 to 10 mm. A maximum decrease of 65.937% in buoyancy factor (B.F) over an equivalent spherical steel pressure hull was computed for carbon/epoxy. Moreover, carbon/epoxy displayed larger decreases in buoyancy factor (B.F) in the case of 4 out of a total of 5 lay-up arrangements. The collapse depth decreased from 517.95 m to 412.596 m for a 5 mm lowest mode imperfection. Similarly, the collapse depth decreased from 522.39 m to 315.6018 for a 5 mm worst mode imperfection.

## 1. Introduction

Due to their beneficial properties: their large specific rigidity, large elastic modulus, enhanced fatigue features, light weight, corrosion resistance, and reduced magnetic and acoustic signatures [1] when compared to metals, Fiber Reinforced Composites (FRCs) are increasingly used in different types of industries. FRCs have also been used in the modeling and fabrication of various shapes pressure hulls including elliptical, cylindrical, spherical and ovoid because of their greater strength-to-weight ratio than metals and other alloys [2]. Many studies are available concerning the optimization of composite pressure hulls featuring cylindrical and elliptical configurations. Nevertheless, as reported in the latest research, spherical and ovoid pressure hulls fabricated with steels and other metal alloys were shown to possess better characteristics than other types of pressure hulls. The spherical hull has a low buoyancy factor and the distribution of stress and displacement in its material is very effective [3], and the most advantageous properties of the ovoid are high strength-to-weight ratio, high span to thickness ratio, good stability, and good material distribution [3,4]. Moreover, the design of composite pressure hulls mainly depends on the number of ply layers, fiber orientation angles and material systems. Therefore, it is very important to conduct optimization studies of the composite spherical pressure hull to further explore the benefits of using composites in its construction.

An inclusive literature survey on the optimization of different composite structures such as beams, plates and shells is given in [5]. Different types of composite pressure hulls were optimized by combining the optimization algorithm with numerical analysis:

Pelletier and Vel [6] used a genetic algorithm (GA) to achieve optimized designs of a graphite/epoxy cylindrical shell. Maximizing the axial and hoop rigidities and minimizing the mass were selected as objectives under material failure constraint. A graphite/epoxy cylindrical shell, where the objective function was the maximization of fundamental natural frequency and buckling load and the ply angle was used as a design variable was optimized by Topal [7] using the Modified Feasible Direction (MFD) method. Messager et al. [8] combined a standard GA with an analytical model of shell buckling to conduct optimization of carbon/epoxy, and glass/epoxy cylindrical pressure hulls for maximum buckling pressure. Shen et al. [9] employed the Sub Problem Approximation (SPA) technique in ANSYS to perform optimization of a composite cylinder for submersible pressure hulls where maximization of design pressure was used as an objective function while buckling pressure and composite failure were used as constraints for the design optimization process. Fathallah et al. [10] employed the Sub Problem Approximation (SPA) method in ANSYS to carry out a design optimization study of stiffened composite elliptical pressure hull to minimize the buoyancy factor (B.F). The material and instability constraints of the optimization were Tsai-Wu and maximum stress composite failure criteria and buckling strength factors. In another study, Fathallah et al. [11] used a similar approach for optimization of a stiffened elliptical composite submerged pressure hull with initial lay-ups [(0°/45°/−45°/90°)4]. The objective function and constraints were the same as used in [10]. Liang et al. [12] employed a Hybrid Genetic Algorithm (HGA) to optimize the PVC sandwich core composite submersible pressure hull. The constraints of the optimization were materials failure and shell buckling under hydrostatic pressure. They used three composite materials, graphite/epoxy, boron/epoxy, and glass/epoxy for modeling the face skin. R. Craven et al. [13] conducted a conceptual design study of a submersible composite pressure hull in ABAQUS. Their study was aimed at reducing the weights of the carbon/epoxy and glass/epoxy composite pressure hulls while keeping composite failure and buckling strength as material and instability constraints.

Shen et al. [14,15], in similar studies, used a GA coupled with numerical analysis for design optimization of carbon/epoxy and carbon/epoxy, boron/epoxy, and glass/epoxy composite pressure hulls under hydrostatic pressure. The objective was to maximize the design pressure under constraints on material failure and buckling instability. The Tsai-Wu composite material failure criterion was used for modeling the composite failure. Li et al. [16] developed a procedure for collaborative design optimization of a ring-stiffened composite pressure hull under material failure constraints. They adopted the ellipsoidal basis function neural network for replacing the finite element (FE) analysis in the optimization program.

Praveen et al. [17] carried out a multi-objective design optimization of a composite cylindrical skirt under the combined effects of axial thrust and torque using a modified NSGA-II. The optimization was carried out for two material systems subjected to material and instability constraints. Wei et al. [18], carried out a stacking sequence optimization for maximum buckling load using a GA coupled with FE analysis. Based on the stiffnesses ratios of the optimized stacking sequence, a stiffness coefficient-based procedure was developed and coupled with the GA to arrive at a stacking sequence close to that previously optimized. Talebitooti et al. [19], developed a technique for a multi-objective design optimization of sound transmission loss for a composite cylindrical shell under a plane sound wave using NSGA-II and First-order Shear Deformation Theory (FSDT). A significant improvement in performance of the structure was obtained based on the maximization of the sound transmission and minimization of the weight. Rouhi et al. [20], performed a buckling optimization study of a variable stiffness composite cylinder subjected to two bending loads at the opposite ends. They employed a compromised programing procedure for the combining of the two objectives (structure performance at the two opposite ends) into a single objective using different weight factors for each objective. Blom et al. [21], conducted a design optimization study of a variable stiffness composite cylinder for maximum buckling pressure subjected to a bending load. The design methodology was implemented in ABAQUS for performing FE analysis. To reduce the analysis time, the FE analysis was then replaced with a surrogate model in the subsequent optimization of the composite cylinder under constraint on material failure. Khani et al. [22] used a semi-analytical analysis coupled with a multi-level optimization approach for design optimization of fiber steered longitudinally stiffened composite cylinders subjected to bending moments. The optimization was carried out for achieving maximum buckling capacity under constraints on the material failure of the composite material. Imran et al. [23], conducted a design optimization study of a submersible composite cylindrical pressure hull for maximum buoyancy factor subjected to constraint on material and buckling strengths. They used several lay-up arrangements and three unidirectional composite materials for modeling of the composite pressure hull. Some studies are also available on the optimization of composite spherical shells. Topal [24], conducted frequency optimization of a composite spherical shell by using the Modified Feasible Direction (MFD) method and FE analysis. The maximization of frequency was used as the objective, and the fiber’s orientation angle was used as a design variable of the optimization study. Faris et al. [25], optimized the ply thickness, fiber’s orientation angle and closed-loop control force of spherical and cylindrical composite shells. The objective was to minimize the dynamic response of the shells under constraints on thickness and control energy. The dynamic response of the shells was represented by the sum of the total elastic energy of the shells and a penalty function of the closed-loop control force.

Several studies looked into the non-linear buckling analysis of composite shells. However, based on the available literature, the research work done on the same subject was mostly restricted to composite cylindrical shells. Several techniques were used for carrying out non-linear buckling analysis and the incorporation methods and influence of different types of imperfections on the buckling behavior of the composite shell were studied in detail. Hilburger et al. [26], carried out experimental and FE investigations to study the influence of imperfections on the buckling performance of graphite/epoxy cylinders under compression loading. They used both traditional and non-traditional imperfection approaches during the non-linear buckling analysis. Tsouvalis et al. [27] performed a numerical and experimental study to investigate the influence of the geometric imperfection on the stability performance of composite cylinders subjected to hydrostatic pressure. The imperfection in the geometry of the composite cylinder was measured by measuring the thickness of the cylinder at 252 points located on the internal and external surfaces. In the FE model, the imperfection on both surfaces was modeled using the two-dimensional smooth cubic spline interpolation. The knockdown factor (KDF) as low as 0.6 was obtained based on the maximum size of the imperfection. Wagner et al. [28] carried out a comprehensive study to calculate the robust knockdown factors (KDFs) of composite cylindrical and conical composite shells under axial load. They modeled the buckling experiments of Starnes et al. [29] using the Single Boundary Perturbation Approach (SBPA) and the Single Perturbation Displacement Approach (SPDA) to demonstrate that these approaches could be efficiently used for the design of composite cylinders subjected to axial loads. They also compared the knockdown factors calculated for axially loaded cylinders from their earlier study, Wagner et al. [30], with those calculated by different empirical approaches for similar cylinders. Castro el al. [31] used geometric imperfection and lower bound approaches to determine the knockdown factors (KDFs) for composite cylinders subjected to axial compression. They compared the Single Perturbation Load Approach (SPLA) with four others routinely used geometric imperfection approaches. FE analysis employing the static analysis with artificial damping was carried out for analyzing the buckling behavior of the composite cylinders up to the post-buckling region. Khakimova et al. [32] used the SPLA to determine the response of the conical composite structures to imperfection. The SPLA was applied to perfect structure and imperfect structure. The geometric imperfection in the structure was included using the thickness imperfection and mid-surface imperfection. It was concluded that the KDFs calculated employing the SPLA was less conservative than those determined by NASA. Wang et al. [33] used the similitude transformation approach and scaling laws to carry out geometrically non-linear buckling analysis of shallow composite spherical shell. The similitude condition and the law for scaling the model was fulfilled by the direct application of the partial similarity transformation to the total energy equation of the system. Hao et al. [34] carried out a numerical investigation on the effects of changing imperfection sizes on the buckling and post-buckling behavior of the stiffened shell of a hyperbolic generatrix shape. Based on the detailed results obtained in the first part, a surrogate optimization study was then carried out to determine the optimum parameters for the stiffened shell under the weight constraints. Wagner et al. [35] developed a new design procedure known as single perturbation cutout approach (SPCA) for the calculation of the KDF for a thin spherical shell. In the new method, the geometric imperfection was replaced with cutouts in the shell. The proposed method was validated against a large volume of available test data and it was also concluded that the SPCA could also be used to assess the imperfection sensitivity of the composite spherical shell subjected to external pressure. Zang et al. [36] employed a third-order shear deformation theory to carry out the non-linear buckling analysis of a laminated shallow spherical shell. The influence of imperfections on the buckling behavior of the composite spherical shell was investigated using a dimple shaped imperfection and a localized flattening on the surface of the shell.

There are no specific rules available in the open literature for carrying out non-linear buckling analysis of spherical pressure hulls constructed with composite materials. However, rules are available for non-linear buckling analysis of shells and pressure hulls constructed with steel and other alloys [37,38]. According to these rules, non-linear buckling analysis of the shells or pressure hulls constructed with steel and other alloys is conducted by using both geometric and material non-linearity with and without the incorporation of imperfection. Appendix E of the Chinese Classification Society Rules for Diving Systems and Submersibles [37] describes the procedure for conducting the non-linear buckling analysis for the determination of the ultimate strength of the cylindrical and spherical pressure hull constructed with steel or other alloys. These rules also state that a non-linear FE analysis of the pressure hull is based on two imperfection analysis methods. In the first method, a linear buckling analysis of the perfect structure is performed to determine the first linear buckling mode. The linear buckling mode is then applied with an imperfection amplitude in the geometric non-linear analysis with material non-linearity and the ultimate strength of the pressure hull is determined. In the second method, which is known as the inherent geometric imperfection method, the imperfection is included in the pressure hull during the modeling process and the non-linear FE analysis is carried out directly to determine the ultimate strength of the pressure hull. In the inherent imperfection method, the imperfection may be included in any position of the spherical pressure hull, while the imperfection is included in the middle section of the cylindrical pressure hull. According to the author’s knowledge non-linear properties of Fiber Reinforced Plastic (FRP) composites such as carbon/epoxy, glass/epoxy and boron/epoxy are not available in the open literature. Therefore, non-linear buckling simulations of the composite spherical pressure hull are performed employing linear elastic material properties with geometric non-linearity and imperfections.

Several studies are available in the literature concerning buckling investigations of non-composite spherical pressure hulls. Both linear and non-linear buckling analyses with geometric and material non-linearity were used in the investigations of spherical hulls [39,40,41,42,43,44,45,46,47,48]. However, the benefit of using composites in the construction of spherical hulls are not yet deeply investigated. In the present paper, an optimization study of a composite spherical pressure hull is performed as an attempt to further reduce its weight as compared to a spherical hull constructed with steels and other alloys. The optimization study is conducted in ANSYS employing a GA coupled with FE analysis [49]. The optimum number of layers and angles of orientation are determined for laminates including, [0_S_/90_T_/0_U_], [10_S_/-10_T_/90_U_/-10_V_/10_W_], [β_1S_/β_2T_], [β_1S_/β_2T_/β_3U_] and [β_1S_/β_2T_/β_3U_/β_4V_/β_5W_] employing carbon/epoxy, glass/epoxy, and boron/epoxy. The minimization of the buoyancy factor (B.F) is selected as the design optimization objective subjected to constraints on Tsai-Wu and Tsai-Hill failure criteria and buckling strength factor. In the second part of this paper, non-linear buckling simulations of the composite spherical pressure hull are conducted to study the effect of imperfection size on the collapse depth of the pressure hull.

## 2. Materials and Methods

### 2.1. Finite Element Model of the Spherical Composite Pressure Hull

The study presented in this paper is a part of our ongoing design optimization study of different types of composite pressure hulls of the same volumes including cylindrical, ovoid, and spherical pressure hulls. The aim of the present research is to determine the decrease in buoyancy factor $\left(D_{BF}\right),\;$compared to a reference steel hull i.e., (weight decrease) on a spherical pressure hull through the use of composite materials as a replacement for HY100 steel.

**Figure 1:**
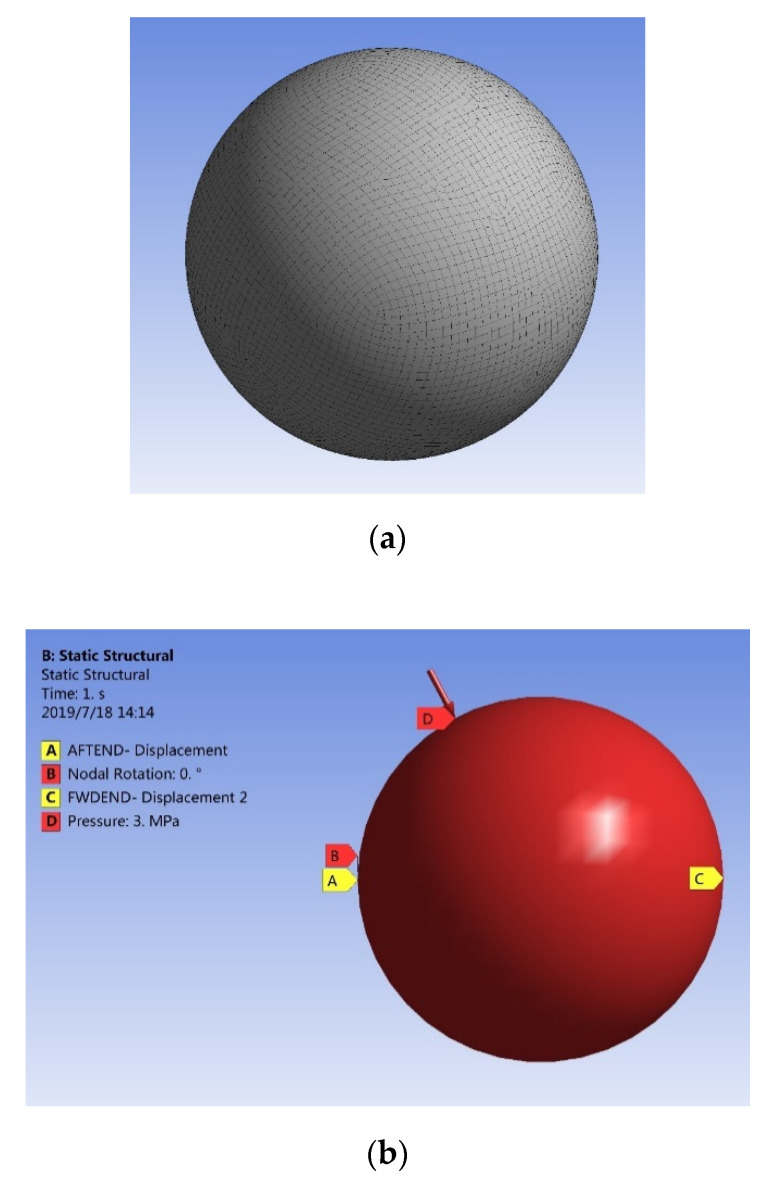
Finite element mesh (**a**) boundary and loading conditions of composite spherical pressure hull (**b**).

The radius of the spherical pressure hull was 2.4245 m. The size selected was based on the cylindrical pressure hull of similar volume with hemispherical ends used in our previous study [23]. The length of the cylindrical section was 8.3 m and radii of both hemispherical ends was 1.37 m. The geometric model of the spherical pressure hull was created employing the primitive feature for a sphere in the geometric modeling tool of ANSYS. The shell elements of ANSYS with an approximate size of 0.1 m. were used to generate the meshed model of the pressure hull. The FE model of the spherical pressure hull is represented in Figure 1a.

Ansys Composite PrePost (ACP) was used to create the composite lay-up of the pressure hull using 0.001 m size ply thickness. In the ACP, a fabric was first selected and the fiber’s orientation and reference directions were created with the help of Rosettes and oriented selection sets. The modeling groups of ACP were then used to create the composite’s plies. The orthotropic material properties used in this paper are listed in Table 1.

It was necessary to apply boundary conditions to both ends to prevent rigid body motion of the spherical hull. The translation in the X, Y and Z directions, and rotational motion in the X direction of one node located at the aft end of the pressure hull was constrained. The translation in the Y and Z directions of one node located at the forward end of the pressure hull was also constrained. These boundary conditions are similar to the boundary conditions employed in the earlier studies on composite cylindrical pressure hulls [13,23]. To represent the effect of hydrostatic pressure, the outer surface of the spherical pressure hull received the application of a uniform pressure of 3 MPa.

The displacement at the aft and forward ends of the pressure hull should be fixed in both Z and Y directions, while the displacement at the node near the center should be fixed at X and Y directions according to the Chinese Classification Society (CCS) Rules for (a) Spherical Hull [37]. To observe the effect of the two types of boundary conditions on the FE analysis results, simulations for both boundary conditions were carried out. The results of the FE analysis for both types of boundary conditions are exactly similar to each other. Therefore, the boundary conditions used in this paper are consistent with the boundary conditions used in [13] and our ongoing study on cylindrical and ovoid composite pressure hulls [23]. The loading and boundary conditions of the pressure hull are represented in Figure 1b.

**Figure 2:**
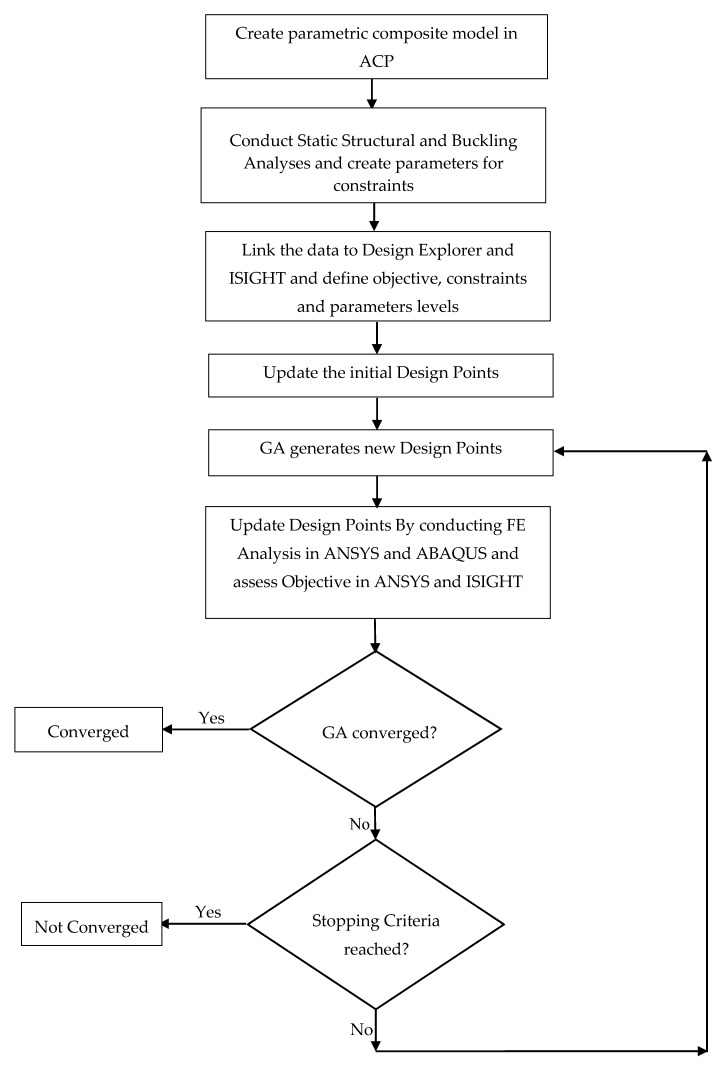
Optimization procedure flow chart [23].

After creating the composite model in the ACP, the data was linked to the Static Structural and Eigenvalue Buckling analysis systems for computing the factors of safety for Tsai-Wu $\left(FS_{TW}\right)$ and Tsai-Hill $\left(FS_{TH}\right)$ failure criteria and buckling strength factors. Similar linear elastic analyses were also used in earlier studies on the conceptual design and design optimization of composite pressure hulls and shell structures [8,9,10,11,12,13,14,16,50,51,52]. After defining the parameters in the relevant analysis systems, the data was then transferred to the Direct Optimization where all input parameters were set and the constraints and objectives were defined. The project was run and the optimized designs of the spherical composite pressure hull were found. The flow chart for the optimization process is presented in Figure 2.

**Figure 3:**
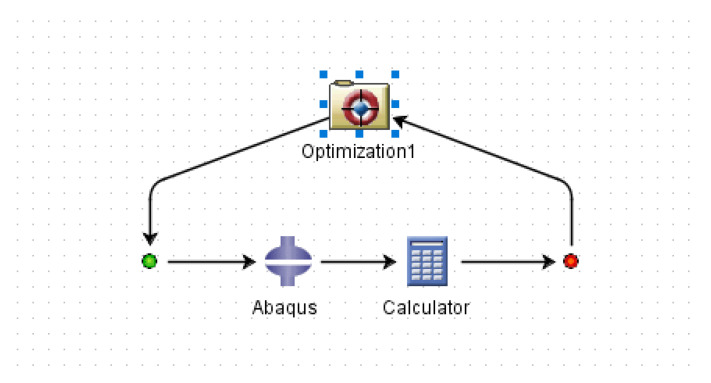
ISIGHT runtime gateway interface.

A parametric design analysis of a reference spherical HY100 steel hull using shell elements was also conducted for comparison purposes. The density of HY100 steel is 7828 Kg/m^3^ and its ultimate yield strength is 790 MPa [53,54]. Furthermore, optimization of one lay-up was also performed in ABAQUS and ISIGHT using NSGA-II. The ISIGHT runtime gateway interface is shown in Figure 3. The NSGA-II in the ISIGHT optimization module is a multi-objective non-dominated sorting genetic algorithm and unlike the multi-objective genetic algorithm of ANSYS, it cannot be used for single objective functions. Therefore, it was necessary to define a second objective to carry out the optimization process. There are three options available for defining the objective functions in the ISIGHT. These options are minimization, maximization and setting a target. In the verification of the optimization process, two objectives were defined in the ISIGHT. One objective was the minimization of the buoyancy factor (B.F) and the other was achieving a target value for the buckling strength factor (λ). The target value for the buckling strength factor (λ) was set around 1.74 based on the values obtained in the design optimization process performed in ANSYS for the same lay-up.

### 2.2. Statement of Design Optimization

The GA used in the present research was a modified form of NSGA-II and used controlled elitism to arrive at a global optimal outcome. It could handle single and multiple objectives and constraints [49,56]. The optimal space filling technique was used to create the initial sampling. The main features of the GA and their corresponding values used in this study are presented in Table 2.

## 3. Results and Discussions

The results of the parametric study for a steel hull are presented in Table 3. The buoyancy factor (B.F) for this hull was 0.17958. For a steel hull of 19 mm thickness, the maximum Von-Mises stress $\left(\sigma_{v}\right)\;$was 368.73 MPa and the buckling strength factor (λ) was equal to 3.1569.

**Figure 4:**
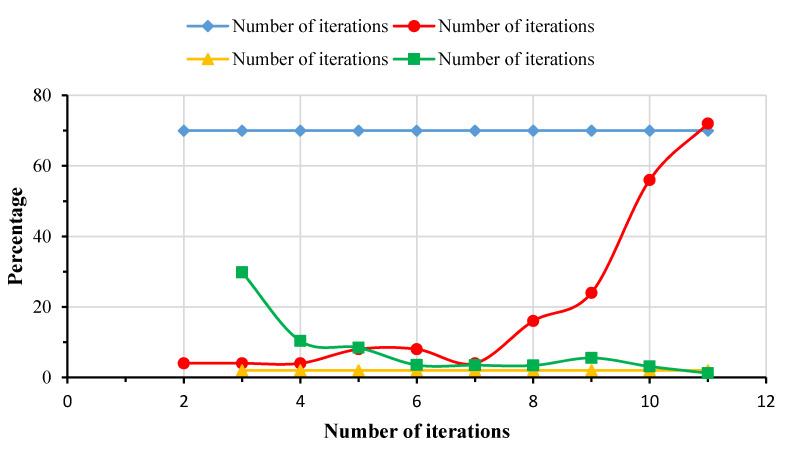
Convergence criteria for optimization of carbon/epoxy lay-up [β_1S_/β_2T_].

**Figure 5:**
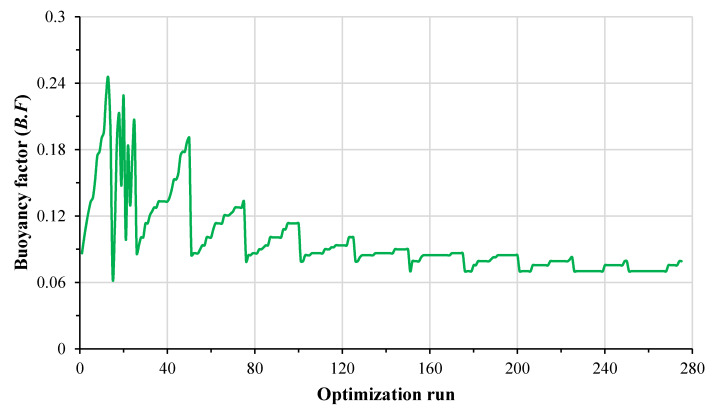
History plot of buoyancy factor (B.F) for optimization of carbon/epoxy lay-up [β_1S_/β_2T_].

The plot of convergence criteria and history plot for the buoyancy factor for a carbon/epoxy lay-up [β_1S_/β_2T_] are shown in Figure 4 and Figure 5. It can be observed from both plots that the optimization of a carbon/epoxy lay-up [β_1S_/β_2T_] converges after fulfilling the 70% Pareto percentage and 2% convergence stability criteria in 275 optimization runs. The comparison between the results of optimization for a carbon/epoxy composite lay-up [0_S_/90_T_/0_U_] using ABAQUS and ISIGHT, and ANSYS is presented in Table 4. It can be noted that the buoyancy factor (B.F) and buckling strength factor calculated using ABAQUS, ISIGHT, and ANSYS optimization studies closely follow each other. The results of design optimization of the spherical composite submersible pressure hull for all considered lay-ups and material systems are discussed in the following sections and listed in Table 5, Table 6, Table 7, Table 8 and Table 9.

In the case of lay-ups [0_S_/90_T_/0_U_] and [10_S_/-10_T_/90_U_/-10_V_/10_W_], the number of each layer could take a value from 1 to 75. The results of lay-up [*0_S_/90_T_/0_U_*] listed in Table 5 demonstrates that the minimum buoyancy factor (B.F) of 0.06117 with $D_{BF}$ equal to 65.937% was computed for carbon/epoxy. The values of $FS_{TW}$, $FS_{TH}$ and λ of carbon/epoxy equaled 4.3119, 2.7085 and 1.7341 respectively. In the case of the glass/epoxy composite, the buoyancy factor (B.F) equaled 0.10143 with a decrease over the reference steel hull $D_{BF}$ = 43.518%. For glass/epoxy, the values of $FS_{TW}$, $FS_{TH}$ and λ were computed as 2.6486, 2.286 and 1.8912 respectively. Similarly, the buoyancy factor (B.F) for boron/epoxy was computed as 0.08452 with a decrease over the reference steel hull $D_{BF}$ = 52.934%. For the same material, the values of $FS_{TW}$, $FS_{TH}$ and λ were computed as 4.7244, 3.8957 and 2.3474 respectively. In the case of lay-up [0_S_/90_T_/0_U_], the material and stability performance of boron/epoxy is better than both carbon/epoxy and glass/epoxy as it gives higher values of $FS_{TW}$, $FS_{TH}$ and λ as compared to them. Moreover, in an earlier study [23], carbon/epoxy and boron/epoxy have also been demonstrated to have maximum decrease in buoyancy factor ($D_{BF})$ and higher material performance in the case of cylindrical pressure hulls modeled with the lay-up [0_S_/90_T_/0_U_]. Similarly, in another study on the composite cylindrical hull modeled with the same lay-up, carbon/epoxy showed more weight reduction as compared to glass/epoxy for comparable stability performance [13].

As can be noted from Table 6, the minimum buoyancy factor (B.F) in the case of lay-up [10_S_/-10_T_/90_U_/-10_V_/10_W_] was computed for carbon/epoxy equaling 0.084553 with a decrease over the reference steel hull $D_{BF}$ = 52.916%. The values of $FS_{TW}$, $FS_{TH}$ and λ of carbon/epoxy were computed as 2.2364, 1.7482 and 2.7296 respectively. The buoyancy factor (B.F) for glass/epoxy is 0.09418 with $D_{BF}$ = 47.555% and its values of $FS_{TW}$, $FS_{TH}$ and λ were determined as 2.03, 1.8142 and 1.7087 respectively. Similarly, the buoyancy factor (B.F) for boron/epoxy is 0.09901 with a decrease over the reference steel hull $D_{BF}$ = 44.865%. For the same material, $FS_{TW}$, $FS_{TH}$ and λ were computed as 3.8816, 3.523 and 3.1574 respectively. The decrease of buoyancy factor over the reference steel hull $\left(D_{BF}\right)$ of glass/epoxy is lower than that of carbon/epoxy and larger than that of boron/epoxy. Moreover, the values of $FS_{TW}$, $FS_{TH}$ and λ for boron/epoxy are greater than their respective values for both carbon/epoxy and glass/epoxy. This demonstrates that the material failure and buckling performance of boron/epoxy is better than that of carbon/epoxy and glass/epoxy. In an earlier study [23] on the composite cylindrical pressure hull modeled with [10_S_/-10_T_/90_U_/-10_V_/10_W_], boron/epoxy was shown to have a greater decrease in weight and larger factors of safety of failure criteria than carbon/epoxy and glass/epoxy with almost similar stability performance.

In the case of lay-ups [β_1S_/β_2T_], β_1S_/β_2T_/β_3U_] and [β_1S_/β_2T_/β_3U_/β_4V_/β_5W_] design optimization was performed for both the number of layers and orientation angles. During optimization, the orientation angle *β* could take a value from 0° to 90° and the number of layers could take a value from 1 to 75. The results of optimization for lay-up [β_1S_/β_2T_] are presented in Table 7. In the case of this lay-up, carbon/epoxy gave the minimum buoyancy factor (B.F) of 0.08275 with a decrease over the reference steel hull $D_{BF}$ = 53.920%. For carbon/epoxy, $FS_{TW}$, $FS_{TH}$ and λ were calculated as 1.914, 1.5361 and 2.5752 respectively. The value of buoyancy factor (B.F) for glass/epoxy was computed as 0.12075 with a decrease over the reference steel hull $D_{BF}$ = 32.759%. For the same material, the values of $FS_{TW}$, $FS_{TH}$ and λ were calculated as 2.0185, 1.7635 and 2.5894 respectively. In the case of boron/epoxy, the buoyancy factor (B.F) equaled 0.08693 with a decrease over the reference steel hull $D_{BF}$ = 51.592%. Similarly, for boron/epoxy, the values of $FS_{TW}$, $FS_{TH}$ and λ were determined as 2.1027, 1.6846 and 2.8753 respectively. In the case of the lay-up [β_1S_/β_2T_], the material failure and buckling performance was almost the same for all the three composites.

For lay-up [β_1S_/β_2T_/β_3U_], the results of design optimization are presented in Table 8. In the case of the same lay-up, the minimum buoyancy factor (B.F) was computed for boron/epoxy which equaled 0.094181 with a decrease over the reference steel hull $D_{BF}$ = 47.554%. The values of $FS_{TW}$, $FS_{TH}$ and λ of boron/epoxy, were calculated as 2.2841, 1.8729 and 2.8591 respectively. In the case of the same lay-up, the buoyancy factor (B.F) of 0.10434 with a decrease over the reference steel hull $D_{BF}$ = 41.897% was computed for carbon/epoxy, while its values of $FS_{TW}$, $FS_{TH}$ and λ were calculated as 1.8877, 1.786 and 5.6293 respectively. In the case of glass/epoxy, the buoyancy factor (B.F) equaled 0.1304 with $D_{BF}$ = 27.386%. For glass/epoxy, $FS_{TW}$, $FS_{TH}$ and λ were calculated as 1.6605, 1.530 and 3.2226 respectively. Moreover, the value of λ for carbon/epoxy was larger than its respective value for both boron/epoxy and glass/epoxy. Similarly, based on the decrease in buoyancy factor (B.F) over the reference steel hull $\left(D_{BF}\right)$, the performance of all three composites was better in lay-up [0_S_/90_T_/0_U_] as compared to their performance in lay-up [β_1S_/β_2T_/β_3U_]. In the case of lay-up [0_S_/90_T_/0_U_], the values of $FS_{TW}$ and $FS_{TH}$ for all three composites were larger than their respective values in lay-up [β_1S_/β_2T_/β_3U_], while all three composites had a greater value of λ in lay-up [β_1S_/β_2T_/β_3U_] as compared to its value in lay-up [0_S_/90_T_/0_U_]. In the case of a cylindrical composite pressure hull [23], the lay-up [0_S_/90_T_/0_U_] was shown to have a better overall performance for all the studied composites as compared to lay-up [β_1S_/β_2T_/β_3U_].

The results of the design optimization for lay-up [β_1S_/β_2T_/β_3U_/β_4V_/β_5W_] are listed in Table 9. For this lay-up the minimum buoyancy factor (B.F) of 0.10974 with a decrease over the reference steel hull $D_{BF}$ = 38.890%, was calculated for carbon/epoxy. For the same material, the values of $FS_{TW}$, $FS_{TH}$ and λ were calculated as 2.2364, 1.7482 and 2.7296 respectively. In the case of the same lay-up, both glass/epoxy and boron/epoxy had the same buoyancy factor (B.F) of 0.11833 with a decrease over the reference steel hull $D_{BF}$ = 34.107%. The values of $FS_{TW}$, $FS_{TH}$ and λ for glass/epoxy were calculated as 2.03, 1.8142 and 1.7087 respectively, while for boron/epoxy these values were calculated as 3.8816, 3.523 and 3.1574. For similar values of $D_{BF}$, the material failure and buckling performance of boron/epoxy is better than that of both carbon/epoxy and glass/epoxy. Based on the decrease in buoyancy factor over the reference steel hull $\left(D_{BF}\right)$, the performance of all three composites is better in lay-up [10_S_/-10_T_/90_U_/-10_V_/10_W_] as compared to their performance in lay-up [β_1S_/β_2T_/β_3U_/β_4V_/β_5W_]. Similarly, in the case of lay-up [10_S_/-10_T_/90_U_/-10_V_/10_W_], the values of $FS_{TW}$ and $FS_{TH}$ for all three composites are larger than their respective values in lay-up [β_1S_/β_2T_/β_3U_/β_4V_/β_5W_], while all three composites have a greater value of λ in lay-up [β_1S_/β_2T_/β_3U_/β_4V_/β_5W_] as compared to its value in lay-up [10_S_/-10_T_/90_U_/-10_V_/10_W_].

## 4. Non-Linear Buckling Analysis

In all the optimization simulations of the spherical composite pressure hull conducted in this paper, Eigenvalue analysis was employed for calculation of the buckling strength factor (λ). Eigenvalue buckling analysis is a linear buckling analysis, in which the structures are assumed to be linear, elastic, and imperfection free. However, the structures are not purely elastic during all situations and also have imperfections due to discrepancies in their material properties and geometries. The structures may also develop defects during the manufacturing process or their applications. Furthermore, at higher pressures (depth) geometric as well as material non-linearity may occur. Hence, it is very important to perform non-linear buckling analysis in addition to linear buckling analysis to precisely model the behavior of structures experiencing the buckling phenomenon.

### 4.1. Procedure for Non-Linear Buckling Analysis

According to the authors’ knowledge based on available literature, there is no specific procedure available for carrying out non-linear analysis involving composite materials. Some studies on the non-linear buckling analysis, which mostly covered composite cylindrical shells, were cited in the present paper’s introduction. In the open literature, guidelines are available which describe the method for performing non-linear buckling analysis of shells modeled with steels or other alloys. As per these guidelines, non-linear buckling analysis of the steel hull is conducted by employing elastic-plastic material properties of steel and geometric non-linearity with and without imperfection. During non-linear buckling analysis, external pressure is applied to the steel hull in increments until the material yields, and the hull buckles and collapses [13,38]. Several procedures for incorporation of imperfection were reported in the existing literature [31,57]. In the present study, the linear buckling mode-shaped imperfection (*LBMI*) method, which was employed in earlier studies on the non-linear buckling analysis of composite shells [13,31,58,59], was employed to examine the effects of imperfection on the collapse depth of the spherical composite pressure hull. In the *LBMI* method, a linear buckling analysis without imperfections in the spherical composite pressure hull is conducted first and the coordinates of the eigenmodes are identified through editing the keywords file. In the second step, the worst buckling mode is applied with a scaling factor to the spherical composite pressure hull through editing the keyword file again. Normally, the lowest buckling mode is considered to be the worst buckling mode. In the case of closed spaced Eigenvalues however, higher modes may give rise to lower buckling loads. Therefore, several buckling modes should be analyzed to determine the worst buckling mode and this mode should be used for further non-linear buckling analysis [60]. In this study, a total of 10 linear buckling modes were analyzed and the worst buckling mode was determined and then used in subsequent non-linear buckling analysis. The optimum carbon/epoxy lay-up [0_12_/90_17_/0_5_] was used for conducting geometrically non-linear buckling analysis with imperfections. A total of six different imperfection sizes of 1, 2, 3, 4, 5, and 10 mm were added to the lowest or first buckling mode shape and to the worst buckling mode shape of the spherical composite pressure hull computed during linear buckling analysis. The worst buckling mode was found to be the 10th buckling mode of the pressure hull. In ABAQUS, the spherical composite pressure hull was stressed with an external pressure of 6 MPa during RIKS analysis with an NGEOM option. The number of increments was set to 100, which means that the 6 MPa pressure was applied in 100 automatically controlled increments.

### 4.2. Results of Non-Linear Buckling Analysis

**Figure 6:**
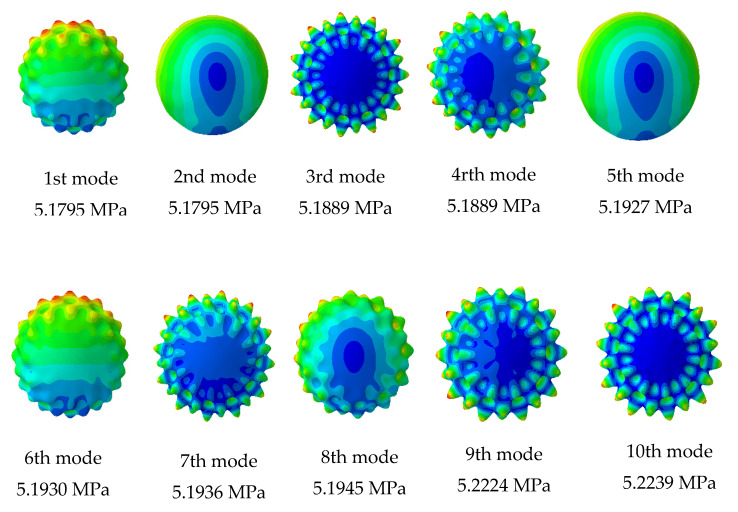
First 10 linear buckling modes of composite submersible spherical pressure hull.

**Figure 7:**
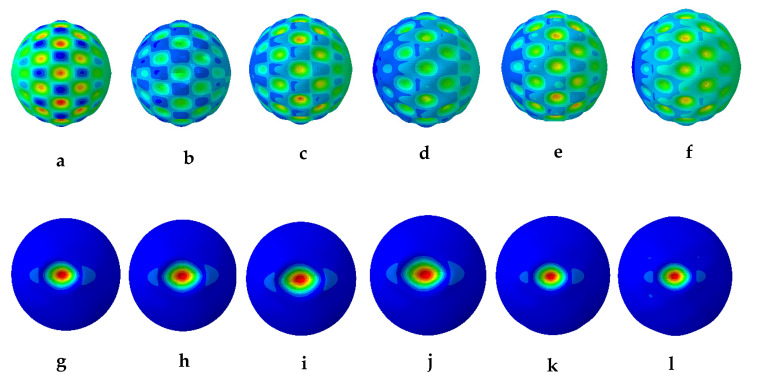
Critical buckling modes (**a**–**f**) and post-buckling modes (**g**–**l**) of the spherical composite pressure hull for 1 mm, 2 mm, 3 mm, 4 mm, 5 mm, and 10 mm size of imperfections in the case of lowest buckling mode imperfection.

**Figure 8:**
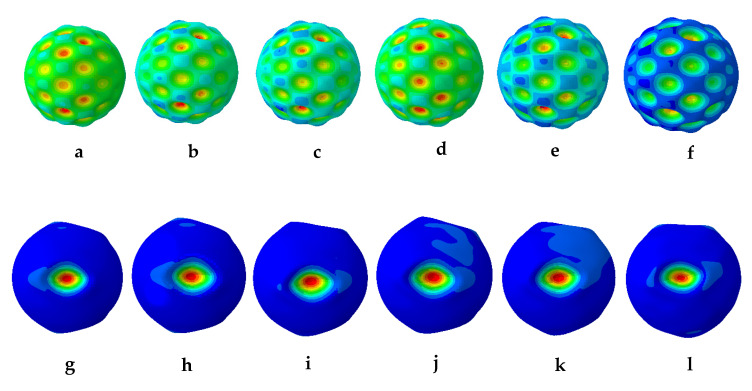
Critical buckling modes (**a**–**f**) and post-buckling modes (**g**–**l**) of the spherical composite pressure hull for 1 mm, 2 mm, 3 mm, 4 mm, 5 mm, and 10 mm size of imperfections in the case of worst buckling mode imperfection.

**Figure 9:**
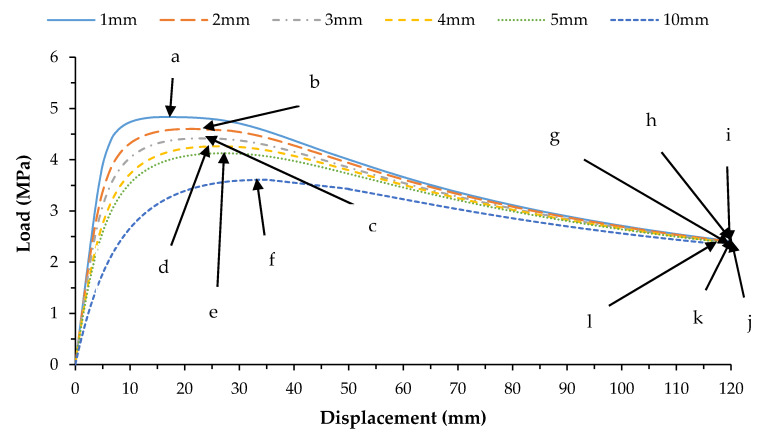
Load-displacement graph for all five imperfections for lowest mode imperfection.

The results from the first 10 linear buckling modes of the composite spherical pressure hull are shown in Figure 6. All linear buckling modes except the 2nd and 5th, show several circumferential waves. The 2nd and 5th buckling modes show none. The critical buckling modes and the post-buckling modes identified during non-linear buckling analysis of the lowest mode shape and of the worst mode shape imperfections for all six imperfection sizes are presented in magnified scales in Figure 7 and Figure 8, respectively. The post-buckling modes in the case of both lowest mode and worst mode imperfections, show deformation in the form of local dents, which are similar to the earlier experimentally identified buckling modes of the carbon/epoxy cylindrical composite pressure hulls reported in [59,61]. The load-displacement relationship obtained from the lowest mode imperfection analysis for all six imperfections is plotted in Figure 9.

**Figure 10:**
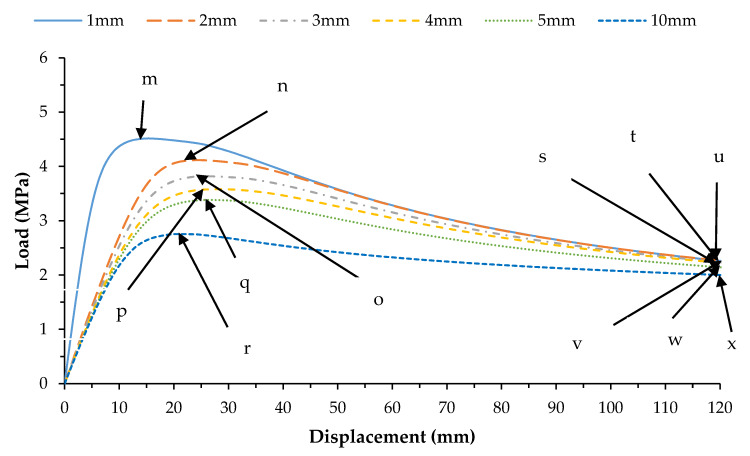
Load-displacement graph for all five imperfections for worst mode imperfection.

In Figure 9, the points (a-f) represent the critical buckling modes and the points (g-l) represent the post-buckling modes of the spherical composite pressure hull. Similarly, the load-displacement relationship obtained from the worst mode imperfection analysis for all six imperfections is plotted in Figure 10. In this figure, the points (m-r) represent the critical buckling modes and the points (s-x) represent the post-buckling modes of the spherical composite pressure hull. The displacement shown in Figure 9and Figure 10 is the inward displacement of the node located at the center of the local dent in each post-buckling mode of the pressure hull.

The results of the imperfection sensitivity study for the lowest mode imperfection and the worst mode imperfection are listed in Table 10 and Table 11, respectively. Failure indices for Tsai-Wu $\left(FI_{TW}\right)$ and Tsai-Hill $\left(FI_{TH}\right)$ failure criteria and the KDFs calculated on the bases of critical buckling pressure of the perfect shell and the critical buckling pressure of the imperfect shell are also computed against each imperfection size and listed in Table 9 and Table 10.

As can be observed from these tables, imperfection size has a profound influence on the collapse depth of the spherical composite pressure hull. In this study, the collapse depth is the depth at which the composite spherical pressure hull first begins yielding. The collapse depth swiftly declines with the rise of imperfection size. In the case of lowest mode imperfection, for a 5 mm imperfection size, the collapse depth decreases from 517.95 m to 412.596 m. Similarly, the imperfection size in the case of worst mode imperfection (Table 11) has a greater effect on the collapse depth as compared to its effect in the case of lowest mode imperfection. At a 5 mm size of imperfection, the collapse depth reduces from 522.39 m to 315.6018 m. Therefore, it is very important to carry out non-linear buckling analysis considering both lowest mode and worst mode imperfections. According to Carven et al., 5 mm imperfections can be tolerated in the design of the composite shell. In the case of worst mode imperfections, the collapse depth is 315.6018 m which is still greater than the 300 m applied depth. Therefore, the optimal design is still considered to be valid after the non-linear buckling analysis. As can be noted from Table 9 and Table 10, the KDFs range from 0.93258 to 0.66093 and from 0.86217 to 0.39888 for the 1 to 10 mm sizes of imperfection in the case of both first mode imperfections and worst mode imperfections, respectively. A similar range of KDFs were obtained in an earlier study on the Z33 composite cylindrical shells for up to a maximum of 5 mm imperfection size using the LBMI method [31]. Moreover, in another study, [27], KDF as small as 0.6 was calculated for composite cylinder. In the present study, for a 5 mm worst mode imperfection the KDF also equals 0.604014.

## 5. Conclusions

This paper describes the design optimization study of the spherical composite pressure hull under hydrostatic pressure employing a genetic algorithm (GA) and finite element (FE) analysis performed in ANSYS. The design optimization study was conducted under constraints on both material failure and buckling instability. A non-linear buckling analysis was also conducted in ABAQUS considering geometric non-linearity and imperfection. Two types of imperfection methods, lowest mode and worst mode, were employed to investigate the effects of imperfection on the collapse depth and overall buckling behavior of the spherical composite pressure hull. The main findings of this research are presented below.

Genetic algorithm (GA) and FE analysis can be employed to identify optimum lay-up arrangements of the spherical composite pressure hull for fabrication or additional examinations such as non-linear buckling analysis and underwater explosion analysis, etc. The optimum designs should be obtained by considering both material failure and buckling instability. Non-linear buckling analysis should also be conducted to investigate the effects of imperfections on the overall buckling behavior of the spherical composite pressure hull.

A maximum decrease of 65.937% in buoyancy factor (B.F) over an equivalent spherical steel hull was measured for carbon/epoxy. Furthermore, carbon/epoxy displayed larger decreases in buoyancy factor (B.F) in the case of 4 out of a total of 5 lay-up arrangements. Boron/epoxy displayed a larger decrease in buoyancy factor (B.F) in the case of 1 out of a total of 5 lay-up arrangements. The glass/epoxy composite showed less overall decrease over an equivalent steel hull in the buoyancy factor as compared to both carbon/epoxy and boron/epoxy.

The collapse depth of the composite spherical submersible pressure hull is deeply affected by the imperfection size. Moreover, the first or lowest buckling mode may not always be the worst buckling mode, especially in the case of closed space Eigenvalues as observed in the present study. Therefore, it is recommended to investigate several linear buckling modes to determine the worst buckling mode for accurately modeling the buckling and post-buckling behavior of the composite spherical pressure hull. In the case of lowest mode imperfection, at the 5 mm imperfection size, the collapse depth was 412.596 m. Similarly, the effect of imperfection size in the case of worst mode imperfection had greater effect on the collapse depth as compared to its effect in the case of lowest mode imperfection. In the case of worst mode imperfection, at the 5 mm size of imperfection, the collapse depth reduced from 522.39 m to 315.6018 m. Moreover, the theoretically calculated KDFs in the present study ranged from 0.93258 to 0.66093 for 1 to 10 mm first or lowest mode imperfections. Similarly, the KDFs ranged from 0.86217 to 0.39888 for 1 to 10 mm worst mode imperfections.

## Figures and Tables

**Table 1:** Material properties of carbon/epoxy, glass/epoxy, and boron/epoxy [10,55].

Properties		Carbon/Epoxy	Glass/Epoxy	Boron/Epoxy
Elastic modulus (GPa)	E_11_	121	45	204
	E_22_	8.6	10	18.5
	E_33_	8.6	10	18.5
	G_12_	4.7	5	5.59
Shear modulus (GPa)	G_13_	4.7	5	5.59
	G_23_	3.1	3.8462	
Poisson’s ratio	ν_12_	0.27	0.3	0.23
	ν_13_	0.27	0.3	0.23
	ν_23_	0.4	0.4	
Density (Kg/m^3^)	*ρ*	1490	2000	2000
Failure stress (MPa)	X_t_	2231	1100	1260
	X_c_	−1082	−675	−2500
	Y_t_	29	35	61
	Y_c_	−100	−120	−202
	Z_t_	29	35	
	Z_c_	−100	−120	
	S_12_	60	80	67
	S_13_	60	80	67
	S_23_	32	46.154	

**Table 2:** Main features of the GA used in this study.

Features	Value
**Size of initial population**	Ten times the number of input parameters
**Number of samples per iteration**	Larger than the half of the size of the initial population
**Crossover**	One point
**Mutation probability**	0.01
**Convergence criteria**	2% and 70% stability convergence and maximum allowable pareto percentages
**Objective of optimization**	$F\left(x\right):\mathrm{Minimize}\;\left[Buoyancy\;factor\;\left(B.F\right)\right]=\frac{Weight\;of\;hull}{Weight\;of\;water\;displaced\;by\;hull}$
**Design variables**	number of ply’s layers and the fiber’s orientation angles
**Material constraints**	$FS_{TW}\geq \;1$ $FS_{TH}\geq \;1$
**Instability constraint**	$\lambda =\frac{P_{cr}}{P}\;\geq 1$
**Side constraints**	$N^{U}\geq \;N_{i}\geq N^{L},\;i=1,2,3\ldots .$ $\beta^{U}\geq {\;\beta }_{i}\geq \beta^{L},\;i=1,2,3\ldots .$

$\;N_{i},$$N^{U}$ and $N^{L}$ represent the number of ply’s layers and its higher and lower bounds for *i*th ply and $\beta_{i},$ $\beta^{U}$ and $\beta^{L}$ represent the orientation angles and its higher and lower bounds for *i*th ply. P and $P_{cr}$ are the applied and critical pressure respectively.

**Table 3:** The results of parametric analysis of the spherical submersible steel hull.

Thickness (mm)	$\sigma_{v}\;(\mathrm{MPa})$	λ	B.F
**19**	368.73	3.1569	0.17958

**Table 4:** Comparison of ANSYS and ISIGHT optimization studies.

Parameters	ANSYS	ISIGHT
Laminate thickness (mm)	34	35
Optimized Lay-up	[0_12_/90_17_/0_5_]	[0_16_/90_17_/0_2_]
$FS_{TW}$	4.3119	2.8766
$FS_{TH}$	2.7085	1.934
λ	1.7341	1.7121
B.F	0.06117	0.06296

**Table 5:** Optimal lay-ups for [0_S_/90_T_/0_U_].

Parameters	Results		
	Carbon/Epoxy	Glass/Epoxy	Boron/Epoxy
**Thickness of ply (mm)**	1	1	1
**Thickness of laminate (mm)**	34	42	35
**Optimized Lay-up**	[0_12_/90_17_/0_5_]	[0_27_/90_14_/0_1_]	[0_27_/90_5_/0_3_]
$FS_{TW}$	4.3119	2.6486	4.7244
$FS_{TH}$	2.7085	2.286	3.8957
λ	1.7341	1.8912	2.3474
B.F	0.06117	0.10143	0.08452
$D_{BF}$ **(%)**	65.937	43.518	52.934

**Table 6:** Optimal lay-ups for [10_S_/-10_T_/90_U_/-10_V_/10_W_].

Parameters	Results		
	Carbon/Epoxy	Glass/Epoxy	Boron/Epoxy
**Thickness of ply (mm)**	1	1	1
**Thickness of laminate (mm)**	47	39	41
**Optimized Lay-up**	[10_27_/-10_7_/90_3_/-10_9_/10_1_]	[10_7_/-10_22_/90_7_/-10_2_/10_1_]	[10_18_/-10_10_/90_3_/-10_9_/10_1_]
$FS_{TW}$	2.2364	2.03	3.8816
$FS_{TH}$	1.7482	1.8142	3.523
λ	2.7296	1.7087	3.1574
B.F	0.084553	0.09418	0.09901
$D_{BF}$ **(%)**	52.916	47.555	44.865

**Table 7:** Optimal lay-ups for [β_1S_/β_2T_].

Parameters	Results		
	Carbon/Epoxy	Glass/Epoxy	Boron/Epoxy
**Thickness of ply (mm)**	1	1	1
**Thickness of laminate (mm)**	46	50	36
**Optimized Lay-up**	[6_19_/65_27_]	[1_22_/47_28_]	[2_33_/53_3_]
$FS_{TW}$	1.914	2.0185	2.1027
$FS_{TH}$	1.5361	1.7635	1.6846
λ	2.5752	2.5894	2.8753
B.F	0.08275	0.12075	0.08693
$D_{BF}$ **(%)**	53.920	32.759	51.592

**Table 8:** Optimal lay-ups for [β_1S_/β_2T_/β_3U_].

Parameters	Results		
	Carbon/Epoxy	Glass/Epoxy	Boron/Epoxy
**Thickness of ply (mm)**	1	1	1
**Thickness of laminate (mm)**	58	54	39
**Optimized Lay-up**	[3_12_/64_26_/33_20_]	[12_4_/18_49_/21_1_]	[1_11_/28_27_/66_1_]
$FS_{TW}$	1.8877	1.6605	2.2841
$FS_{TH}$	1.786	1.530	1.8729
λ	5.6293	3.2226	2.8591
B.F	0.10434	0.1304	0.094181
$D_{BF}$ **(%)**	41.897	27.386	47.554

**Table 9:** Optimal lay-ups for [β_1S_/β_2T_/β_3U_/β_4V_/β_5W_].

Parameters	Results		
	Carbon/Epoxy	Glass/Epoxy	Boron/Epoxy
**Thickness of ply (mm)**	1	1	1
**Thickness of laminate (mm)**	61	49	49
**Optimized Lay-up**	[5_41_/45_9_/15_6_/60_4_/63_1_]	[16_2_/18_11_/18_27_/59_4_/22_5_]	[13_18_/66_19_/51_6_/61_5_/17_1_]
$FS_{TW}$	2.0898	1.6128	2.6795
$FS_{TH}$	1.6369	1.4857	2.2619
λ	6.1479	2.7678	6.8487
B.F	0.10974	0.11833	0.11833
$D_{BF}$ **(%)**	38.890	34.107	34.107

**Table 10:** Effect of imperfection on the collapse depth for lowest mode imperfection.

Imperfection Size (mm)	$FI_{TW}\;$	$FI_{TH}\;$	Collapse Depth (m)	KDF
0	0.7815	0.946	517.95	1
1	0.8447	1.025	483.0336	0.9325
2	0.7968	0.9653	459.561	0.8872
3	0.8784	1.0540	440.8362	0.8511
4	0.8780	1.0370	422.7414	0.8161
5	0.8449	1.0030	406.4436	0.7847
10	1.0910	1.0710	342.3330	0.6609

**Table 11:** Effect of imperfection on the collapse depth for worst mode imperfection.

Imperfection Size (mm)	$FI_{TW}\;$	$FI_{TH}\;$	Collapse Depth (m)	KDF
0	0.7815	0.946	522.390	1
1	0.8093	0.9815	450.4434	0.8621
2	0.8301	0.9711	404.3784	0.7740
3	0.9244	1.0300	371.6622	0.7113
4	1.0190	1.0310	342.6348	0.6558
5	1.0390	1.1025	315.6018	0.6041
10	0.9488	0.9556	208.3722	0.3988
